# Long non‐coding RNA cardiac hypertrophy‐associated regulator governs cardiac hypertrophy via regulating miR‐20b and the downstream PTEN/AKT pathway

**DOI:** 10.1111/jcmm.14641

**Published:** 2019-08-29

**Authors:** Mingyu Zhang, Yuan Jiang, Xiaofei Guo, Bowen Zhang, Jiangjiao Wu, Jiabin Sun, Haihai Liang, Hongli Shan, Yong Zhang, Jiaqi Liu, Ying Wang, Lu Wang, Rong Zhang, Baofeng Yang, Chaoqian Xu

**Affiliations:** ^1^ Department of Pharmacology (State‐Province Key Laboratories of Biomedicine‐Pharmaceutics of China, Key Laboratory of Cardiovascular Medicine Research, Ministry of Education) College of Pharmacy Harbin Medical University Harbin China; ^2^ Department of Pharmacy The Second Affiliated Hospital of Harbin Medical University Harbin China; ^3^ Center of Chronic Diseases and Drug Research of Mudanjiang Medical University of Alliance of Sino‐Russian Medical Universities Mudanjiang Medical University Mudanjiang China; ^4^ Department of Urology The Fourth Hospital of Harbin Medical University Harbin China

**Keywords:** cardiac hypertrophy, cardiac hypertrophy‐associated regulator, long non‐coding RNA, miR‐20b, phosphatase and tensin homolog

## Abstract

Pathological cardiac hypertrophy (CH) is a key factor leading to heart failure and ultimately sudden death. Long non‐coding RNAs (lncRNAs) are emerging as a new player in gene regulation relevant to a wide spectrum of human disease including cardiac disorders. Here, we characterize the role of a specific lncRNA named cardiac hypertrophy‐associated regulator (CHAR) in CH and delineate the underlying signalling pathway. CHAR was found markedly down‐regulated in both in vivo mouse model of cardiac hypertrophy induced by pressure overload and in vitro cellular model of cardiomyocyte hypertrophy induced by angiotensin II (AngII) insult. CHAR down‐regulation alone was sufficient to induce hypertrophic phenotypes in healthy mice and neonatal rat ventricular cells (NRVCs). Overexpression of CHAR reduced the hypertrophic responses. CHAR was found to act as a competitive endogenous RNA (ceRNA) to down‐regulate miR‐20b that we established as a pro‐hypertrophic miRNA. We experimentally established phosphatase and tensin homolog (PTEN), an anti‐hypertrophic signalling molecule, as a target gene for miR‐20b. We found that miR‐20b induced CH by directly repressing PTEN expression and indirectly increasing AKT activity. Moreover, CHAR overexpression mitigated the repression of PTEN and activation of AKT by miR‐20b, and as such, it abrogated the deleterious effects of miR‐20b on CH. Collectively, this study characterized a new lncRNA CHAR and unravelled a new pro‐hypertrophic signalling pathway: lncRNA‐CHAR/miR‐20b/PTEN/AKT. The findings therefore should improve our understanding of the cellular functionality and pathophysiological role of lncRNAs in the heart.

## 1 INTRODUCTION

Cardiac hypertrophy (CH) is an adaptive response to pathological pressure overload or neurohormonal stimulation to reduce wall stress by increasing wall thickness and to maintain normal cardiac function. However, prolonged CH can develop into heart failure (HF) and eventually lead to sudden death.^1^, ^2^, ^3^ CH is accompanied by increases in cardiomyocyte size, protein synthesis and expression of many foetal genes.^4^, ^5^ Nevertheless, the underlying molecular mechanisms of CH are still incompletely understood.

MicroRNAs (miRNAs) are a class of non‐coding RNAs of about 22 nucleotides long, and they primarily act as negative regulators of genes by binding to the complementary 3′ untranslated region.^6^, ^7^ Previous studies have confirmed that miRNAs play a significant role in the regulation of cell differentiation, proliferation, and apoptosis.^8^, ^9^ MiRNAs participate in regulating cardiac function, several of which have been reported as important regulators of CH and HF.^10^, ^11^, ^12^ For example, miR‐22 induces CH,^13^ miR‐208 protects against CH,^14^ and miR‐155 regulates CH by targeting BRCA1.^15^

Phosphatase and tensin homolog deleted on chromosome ten (PTEN) is one of the familiar mutated tumour suppressors. PTEN is a negative regulator of the PI3K/AKT signal pathway, and it critically determines ischaemic cardiomyocyte apoptosis, left ventricular remodelling caused by myocardial infarction, ischaemia reperfusion injury and cardiac contractile dysfunction.^16^, ^17^, ^18^, ^19^ It has been demonstrated that PTEN inhibits CH by modulating actin dynamics.^20^ On the other hand, miR‐20b has been reported to directly regulate PTEN expression in breast cancer cells.^21^

Long non‐coding RNAs (lncRNAs) have no significant protein‐coding potential and normally have more than 200 nucleotides in length. They are distributed in both nucleus and cytoplasm, and regulate the intracellular signalling pathways via different mechanisms including chromatin modification,^22^ gene transcriptional regulation^23^, ^24^ and competitive endogenous RNA (ceRNA).^25^ lncRNAs can be classified as sense, antisense, bidirectional, intronic and intergenic lncRNAs.^26^, ^27^ A conservative estimate suggests that human genome and mouse genome contain about 25 000 and 30 000 putative lncRNAs transcripts, respectively.^28^ Recent studies have revealed that cardiac hypertrophy‐related factor (CHRF) regulates CH by targeting miR‐489,^29^ nuclear factor of activated T cells (NFAT) accelerates the development of CH by regulating Chast (cardiac hypertrophy‐associated transcript),^30^ and Chaer (cardiac hypertrophy‐associated epigenetic regulator) regulates CH as an epigenetic checkpoint.^31^ Yet, given the large number of lncRNAs in a cell or an organism, our current understanding of the biological function and pathophysiological roles of lncRNAs is still rather limited.

We have previously characterized the lncRNA expression signature using microarray analysis in conjunction with real‐time RT‐PCR methods in a mouse model of CH induced by pressure overload after transverse aortic constriction (TAC) and identified a particular lncRNA AK134605 with its expression robustly down‐regulated in CH.^32^ We have also demonstrated that the expression of miR‐20b was significantly up‐regulated in CH.^11^ The objectives of the present study were to examine whether AK134605 plays a role in CH and, if yes, then to decipher the underlying mechanisms for the action of this lncRNA in CH.

## 2 MATERIALS AND METHODS

### 2.1 Animals

C57BL/6 male mice weighing 20‐25 g were obtained from the Liaoning Changsheng Biotechnology Co. Ltd. The animals were kept under standard animal room conditions (temperature 21 ± 1°C; humidity 55%‐60%) with food and water continuously available for 1 week before the experiments. All experimental procedures were conformed to the NIH guidelines and were approved by the Institutional Animal Care and Use Committee of Harbin Medical University.

### 2.2 Pressure‐overload cardiac hypertrophy

The pressure‐overload model of cardiac hypertrophy was created by transverse aortic constriction (TAC). Adult mice (C57BL/6), weighing 20‐25 g, were anaesthetized with amobarbital (60 mg/kg, intraperitoneal injection). The adequacy of anaesthesia was judged by the absence of withdrawal reflex to tail pinch. Mice were placed in the supine position. After successful endotracheal intubation, the cannula was connected to a volume cycled rodent ventilator (Ugo Basile SRL 3). Chest was opened to identify the thoracic aorta. A 5‐0 silk suture was placed through below the transverse aorta and tied around a 26‐gauge blunt needle which was subsequently removed. The chest was closed, and the animals were kept under ventilation until autonomic breath was successfully recovered. The velocity of blood flow within the banding site of aorta was monitored using Visual Sonic Ultrasound system (Vevo 2100). Animals were checked daily for signs of pain or distress, and buprenex at 0.05 mg/kg SQ was given before and every 12 hours for 48 hours. After 4 weeks, survived animals were analysed with echocardiography and then killed by cervical dislocation. Hearts were quickly excised, cleaned in PBS and weighted. They were then kept in 4% paraformaldehyde or dissected into left ventricle (LV), right ventricle and ventricular septum, rapidly frozen in liquid nitrogen and stored at −80°C for subsequent analyses. Tibia length was measured as a comparison for varying weights of the hearts.

### 2.3 Construction of lentivirus vectors

The lentivirus vectors carrying a short interference RNA to CHAR (Lenti‐sh‐CHAR) for loss‐of‐function or a scramble fragment (Lenti‐sh‐Scr) for negative control, and the lentivirus vectors carrying the full‐length sequence to CHAR (Lenti‐CHAR) for gain‐of‐function or empty vector (Lenti‐Vector) for negative control were constructed by Cyagen Biosciences. Virus titre was 1 × 10^9^ U/mL. C57BL/6 mice were injected through left ventricular chamber for three weeks before TAC surgery.

### 2.4 Histological analysis

The hearts were fixed with 4% paraformaldehyde, embedded in paraffin, sectioned into 7‐μm slices and subjected to standard haematoxylin and eosin (H&E) staining. The slices were visualized under a microscope (Nikon 80i).

### 2.5 Echocardiography

Mice were lightly anaesthetized with amobarbital (60 mg/kg, intraperitoneal injection) and then placed on a platform. Cardiac anatomical and functional parameters were evaluated by two‐dimensional transthoracic echocardiography using a Visual Sonic Ultrasound system (Vevo 2100). The heart was imaged in a parasternal short‐axis view at the level of the papillary muscles to record M‐mode measurements and to determine heart rate, wall thickness, and end‐diastolic and end‐systolic dimensions. LV wall thickness was used as an index of cardiac hypertrophy.

### 2.6 Culturing and treatment of neonatal rat ventricular cells

The procedures for culturing NRVCs were essentially the same as previously described.^12^ Rats of 1‐ to 3‐day‐old were anaesthetized using 4%‐5% isoflurane‐inhalation anaesthesia, and adequate anaesthesia was assured by the absence of reflexes. The animals were killed by dislocation of the neck, prior to rapid heart excision. The cells were plated into a 6‐well plate at a density of 3 × 10^5^ cells per well and incubated at 37°C in humidified air with 5% CO_2_ for 48 hours, and 0.1 mmol/L bromodeoxyuridine (Sigma) was then added into the medium to deplete non‐cardiomyocytes. Prior to the experimental measurements, the cells were starved for 12 hours and then treated with varying agents.

### 2.7 Cell culture

Neonatal rat ventricular cells were cultured in humidified air with 5% CO_2_ at 37°C for 48‐72 hours., Then, the cells were treated with angiotensin II (AngII; Sigma) at 200 nmol/L for 48 hours or Akt inhibitor MK2206 (Selleck) at 100 nmol/L for 48 hours.

### 2.8 Western blot

The concentration of proteins purified from LV or cultured NRVCs was determined with BCA Protein Assay Kit. The samples were subjected to electrophoresis in 10% SDS‐PAGE and then transferred to nitrocellulose filter membrane. The membrane was blocked in 5% skim milk at 25°C for 1.5 hours and then incubated with the primary antibodies. The anti‐PTEN antibody (1:1000; Abcam), anti‐phospho‐Akt (Ser473) antibody (1:2000; Cell Signaling), anti‐Akt (pan) antibody (1:1000; Cell Signaling) and GAPDH (1:2000; Proteintech) were used in this study. After washing four times with PBS, the membrane was incubated with the fluorescence‐conjugated secondary antibody (Invitrogen) at 1:8000 dilution for 1 hour. Western blot bands were quantified by using Odyssey infrared imaging system (LI‐COR).

### 2.9 MiRNA, siRNA and plasmid transfection

miR‐20b mimic, negative control miRNA (NC), miR‐20b antisense inhibitor (AMO‐20b), CHAR small interference RNA (siRNA) and a non‐related, scrambled RNA fragment without any match to the mouse genomic sequence used as a control were synthesized by RiboBio. CHRF overexpression plasmid was constructed by Generay. NRVCs were transfected with miR‐20b, NC, AMO‐20b, siRNA or plasmid using X‐treme (Roche) following the manufacturer's protocol.

### 2.10 Quantitative real‐time PCR

Total RNA samples were extracted from cultured NRVCs and cardiac tissue using TRIzol reagent (Invitrogen). To detect mRNA expression levels of lncRNA CHRF, miR‐20b, PTEN and hypertrophic biomarkers ANP, BNP and β‐MHC, real‐time PCR was carried out on ABI 7500 fast real‐time PCR system (Applied Biosystems) with SYBR Green I (Applied Biosystems). GAPDH was used as an internal control. miR‐20b level was quantified by the mirVana qRT‐PCR miRNA Detection Kit (Ambion) according to the manufacturer's protocols and as previously described.^33^, ^34^ U6 was used as an internal control for miRNA quantification. The primer pairs used in this study are as follows:

- ANP forward 5′‐CTCCGATAGATCTGCCCTCTTGAA‐3′,
- ANP reverse 5′‐GGTACCGGAAGCTGTTGCAGCCTA‐3′;
- BNP forward 5′‐TGATTCTGCTCCTGCTTTTC‐3′,
- BNP reverse 5′‐GTGGATTGTTCTGGAGACTG‐3′;
- β‐MHC forward 5′‐CCAGAAGCCTCGAAATGTC‐3′,
- β‐MHC reverse 5′‐CTTTCTTTGCCTTGCCTTTGC‐3′;
- PTEN forward 5′‐CCAGTCAGAGGCGCTATGTA‐3′,
- PTEN reverse 5′‐TCCGCCACTGAACATTGGAA‐3′;
- CHAR forward 5′‐GATCATGGCCGTGAGTGT‐3′,
- CHAR reverse 5′‐GGAGTGAAGCGGTGGTAT‐3′;
- miR‐20b forward 5′‐CAAAGTGCTCATAGTGC‐3′,
- miR‐20b reverse 5′‐TGTCGTGGAGTCGGCAATT‐3';
- and miR‐20b primer for reverse transcription: 5′‐ GTCGTATCCAGTGCGTGTCGTGGAGTCGGCAATTGCACTGGATACGACCTACCT‐3′.

### 2.11 Measurement of cell surface area

Cell surface area was measured as previously described in detail.^11^ Briefly, cardiomyocytes were fixed with 4% paraformaldehyde for 20 minutes, penetrated by 0.4% Triton X‐100 for 1 hour and then blocked by goat serum at 37°C for 1 hour. The cells were first incubated with anti‐sarcomeric alpha actinin antibody (1:250; Abcam) at 4°C overnight and subsequently with a DyLight 594 goat anti‐mouse antibody at room temperature for 1 hour. Then, the cells were incubated with DAPI for 7 minutes. Immunofluorescence was visualized under a fluorescence microscope (Nikon 80i). Cell surface area was measured by Image‐Pro Plus Data Analysis Software and quantified by measuring 50 randomly selected cells from each of three independent experiments. The measured values were averaged for statistical analyses.

### 2.12 Protein/DNA ratio

After standard culture procedures, the ratio of total protein to DNA of cardiomyocytes was analysed. In brief, cells were rinsed in cold PBS twice and scraped with 100 μL of lysis buffer (150 mmol/L NaCl, 15 mmol/L sodium citrate and 0.25% SDS; pH = 7.0). The collected cells were immediately frozen and stored at −20°C. Then samples were thawed and vortexed, and 1 μL sample was applied to determine the total protein content using BCA method. DNA concentrations were detected by using a DNA Quantification Kit (Sigma‐Aldrich).

### 2.13 Statistical analysis

Data are presented as means ± SEM. The statistical comparisons among multiple groups were performed with analysis of variance (ANOVA). If significant effects were indicated by ANOVA, a *t* test using the Bonferroni correction or a Dunnett's test was used to evaluate the significance of the differences between the individual means. Otherwise, the data were compared by Student's *t* test. A two‐tailed difference with *P* < .05 was considered statistically significant. The data were analysed using GraphPad Prism 5.0.

## 3 RESULTS

### 3.1 Establishment of in vivo and in vitro models of cardiac hypertrophy

Cardiac hypertrophy was created by TAC‐induced pressure overload in C57BL/6 mice. After four weeks of TAC, echocardiography analysis demonstrated significant thickening of LV wall in CH mice compared with the sham‐operated control mice (Figure S1A). The ejection fraction (EF) and left ventricular fractional shortening (FS) were both declined in TAC mice relative to the sham‐operated control animals (Figure S1B,C). The values of heart weight/body weight and heart weight/tibia length in the TAC group were higher than the sham counterparts (Figure S1D,E). H&E staining of cardiac sections showed that the cross‐sectional area was substantially enlarged in TAC mice (Figure S1F). The mRNA levels of hypertrophic biomarkers atrial natriuretic peptide (ANP), brain natriuretic peptide (BNP) and β‐myosin heavy chain (β‐MHC) were up‐regulated in TAC mice (Figure S1G). The same results were reproduced in cultured NRVCs exposed to AngII at a concentration of 200 nmol/L for 48 hours to induce cardiomyocyte hypertrophy. As depicted in Figure S1H,I, cell size was remarkably enlarged; meanwhile, the mRNA levels of ANP, BNP and β‐MHC were also markedly up‐regulated.

### 3.2 CHAR participates in pathological cardiac hypertrophy in vitro

**Figure 1:**
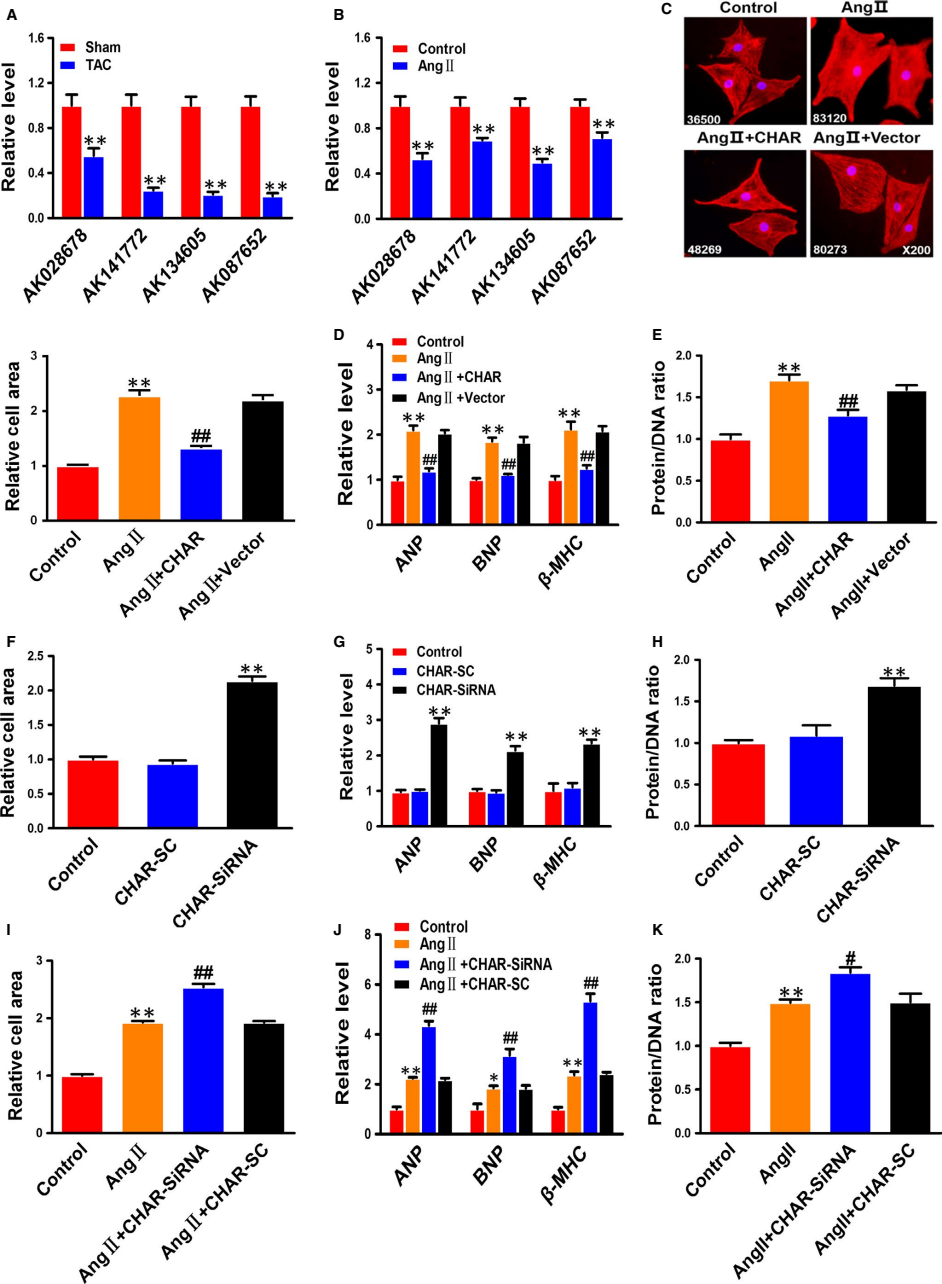
The anti‐hypertrophic effects of cardiac hypertrophy‐associated regulator CHAR in NRVCs. A, Down‐regulation of four lncRNAs in mice with CH induced by TAC compared with that in sham‐operated animals. The four lncRNAs tested were selected based on our previous microarray analysis.^32^ **P < .01 vs Sham; n = 10. B, Expression down‐regulation of four selected lncRNAs in NRVCs treated with AngII. **P < .01 vs Control; n = 10. C, CHAR overexpression reversed the enlarged cell size induced by AngII in NRVCs, n = 50. D, CHAR overexpression reversed the increased mRNA levels of ANP, BNP and β‐MHC induced by AngII in NRVCs (n = 4). E, CHAR overexpression reversed the increased protein/DNA ratio (n = 6) induced by AngII in NRVCs. **P < .01 vs Control, and ^##^ *P *< .01 vs AngII. F, Knockdown of CHAR by siRNA enlarged cell size in NRVCs (n = 50), resembling the hypertrophic cell growth. G, Knockdown of CHAR by siRNA increased the mRNA levels of the hypertrophic marker genes ANP, BNP and β‐MHC in NRVCs (n = 5). H, Knockdown of CHAR by siRNA increased the protein/DNA ratio in NRVCs (n = 4). SC: the scrambled negative control RNA. **P < .01 vs CHAR‐SC. I‐K, Knockdown of CHAR by siRNA exacerbated the hypertrophic responses induced by AngII in NRVCs with enlarged cell size (n = 50; I); elevated mRNA levels of ANP, BNP and β‐MHC (n = 4; J); and increased protein/DNA ratio (n = 4; K). **P < .01 & *P < .05 vs Control; ^##^ *P *< .01 & ^#^ *P *< .05 vs AngII. AngII, angiotensin II; ANP, atrial natriuretic peptide; BNP, brain natriuretic peptide; CH, cardiac hypertrophy; CHAR, cardiac hypertrophy‐associated regulator; lncRNA, Long non‐coding RNA; NC, negative control; NRVC, neonatal rat ventricular cells; PTEN, Phosphatase and tensin homolog; β‐MHC, β‐myosin heavy chain

To explore the potential role of lncRNAs in CH, we first conducted quantitative PCR on four lncRNAs (AK134605, AK028678, AK141772 and AK087652) that had been found to be down‐regulated in our previous microarray analysis.^32^ AK134605 was found remarkably down‐regulated (Figure 1A), a result consistent with the finding in our published study.^32^ In agreement with the in vivo experiments described above, the level of AK134605 was also considerably down‐regulated in AngII‐treated NRVCs (Figure 1B). We therefore choose to study AK134605 in detail for it was the most down‐regulated lncRNA in both in vivo and in vitro models among the four lncRNAs examined. For convenience, we named AK134605 cardiac hypertrophy associated regulator (CHAR) in the rest of our manuscript.

To clarify whether down‐regulation of CHAR really plays a role in pathologic hypertrophy or is merely a bystander of CH, we first transfected CHAR plasmid for overexpression or CHAR siRNA into NRVCs for silencing endogenous CHAR. The transfection efficiency was verified by a significant increase in CHAR level with the CHAR‐plasmid and a significant decrease in endogenous CHAR with siRNA (Figure S2A‐D). Immunostaining for α‐actinin displays that CHAR overexpression diminished the enlargement of cardiomyocyte size induced by AngII (Figure 1C). Similarly, the AngII‐induced increases in the expression of ANP, BNP and β‐MHC, as well as the increased protein/DNA ratio, were significantly mitigated by CHAR compared with mock‐treated NRVCs (Figure 1D,E). Most notably, transfection of CHAR siRNA into normal NRVCs increased cell size; mRNA levels of ANP, BNP and β‐MHC; and the protein/DNA ratio (Figure 1F‐H). Silence of CHAR by its siRNA exacerbated the hypertrophic phenotypes in NRVCs in the presence of AngII, as indicated by significant aggravation of AngII‐induced increases in cell size, mRNA levels of CH‐marker genes and the protein/DNA ratio (Figure 1I‐K). These data indicated that CHAR critically participates in the regulation of CH as an anti‐hypertrophic lncRNA.

### 3.3 CHAR regulates cardiac hypertrophy in vivo

**Figure 2:**
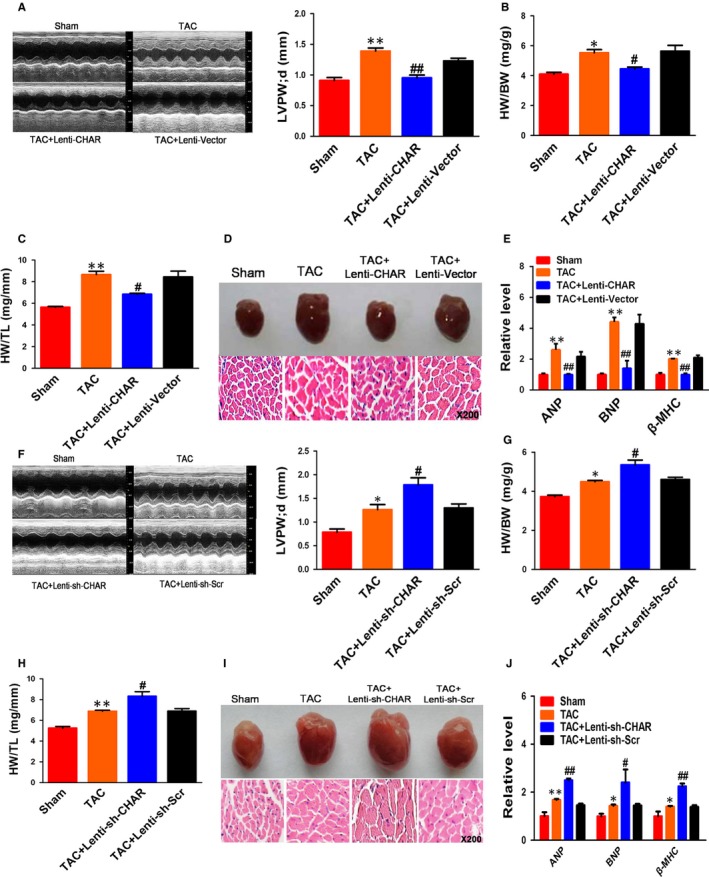
CHAR regulates cardiac hypertrophy in a mouse TAC model. A, Echocardiography showing the attenuation of hypertrophic responses by CHAR overexpression with Lenti‐CHAR in TAC mice relative to sham‐operated control mice. **P < .01 vs sham; ^##^ *P *< .01 vs TAC + Lenti‐Vector; n = 6. B & C, Decreases in the ratios of HW/BW and HW/TL by Lenti‐CHAR for CHAR overexpression in TAC mice relative to sham‐operated control mice. **P < .01 & *P < .05 vs sham; ^#^ *P* < .05 vs TAC + Lenti‐Vector n = 6. D, Lenti‐CHAR substantially attenuated haematoxylin and eosin staining of myocardium in TAC mice relative to TAC mice without CHAR overexpression. E, Decreases in mRNA levels of the hypertrophic biomarkers ANP, BNP and β‐MHC in TAC mice injected with Lenti‐CHAR relative to sham counterparts. **P < 0.01vs Sham; ^##^ *P* < .01 & ^#^ *P* < .05 vs TAC + Lenti‐Vector. n = 4. F‐J, Knockdown of CHAR by shRNA (sh‐CHAR) exacerbated the hypertrophic responses induced by TAC: enlarged LV wall (n = 5; F), increased ratios of HW/BW and HW/TL (n = 5; G‐H), enlarged cardiac muscles (I) and elevated mRNA levels of ANP, BNP and β‐MHC (n = 4; J) **P < .01 & *P < .05 vs sham; ^##^ *P *< .01 & ^#^ *P* < .05 vs TAC + Lenti‐sh‐Scr. ANP, atrial natriuretic peptide; BNP, brain natriuretic peptide; CHAR, cardiac hypertrophy‐associated regulator; TAC, transverse aortic constriction β‐MHC, β‐myosin heavy chain

To better understand the function of CHAR in the heart, mice were injected through left ventricular chamber with lentivirus vector carrying the full‐length sequence of CHAR or the empty vector (Vector) as a control for three weeks, followed by TAC for four weeks. Real‐time PCR (RT‐PCR) analysis confirmed that CHAR expression was significantly increased (Figure S2E). Echocardiography analysis showed that LV wall was thinner in CHAR‐overexpressing mice than in TAC mice treated with empty vector (Figure 2A). EF and FS in CHAR‐overexpressing TAC mice were greater than in vector‐treated TAC mice (Figure S3A,B). There was no significant change in heart rate between CHAR‐overexpressing TAC mice and sham‐operated control mice (Table S2). CHAR‐overexpressing mice exhibited lower ratios of heart/body weight and heart weight/tibia length (Figure 2A,B). Moreover, H&E staining showed that the cross‐sectional area in CHAR‐overexpressing mice was decreased compared with the sham control mice (Figure 2D). As shown in Figure 2E, mRNA levels of ANP, BNP and β‐MHC in CHAR‐overexpressing TAC mice were down‐regulated relative to those in vector‐treated TAC mice.

Meanwhile, we tested whether CHAR knockdown alone could affect CH phenotypes. We injected lentivirus carrying CHAR short RNA (sh‐CHAR) or scramble fragment into left ventricular chamber for three weeks before TAC surgery. We found that CHAR was markedly decreased by sh‐CHAR (Figure S2F). Echocardiography analysis demonstrated that LV wall was significantly thickened in TAC mice treated with sh‐CHAR compared with the TAC mice treated with scramble fragment (Figure 2F). EF and FS were both declined in CHAR‐silenced TAC mice relative to the sham‐operated control animals (Figure S3C,D). We also measured heart rate and found no significant change between sh‐CHAR TAC mice and sham‐operated control mice (Table S1).

The ratios of heart weight/body weight and heart weight/tibia length in the sh‐CHAR group were higher than in the scramble group (Figure 2G,H). H&E staining revealed a significant increase in the cross‐sectional area of ventricular myocardium from sh‐CHAR mice after 4 weeks of TAC surgery (Figure 2I). The mRNA levels of hypertrophic biomarkers ANP, BNP and β‐MHC were significantly up‐regulated in sh‐CHAR mice compared to scramble mice (Figure 2J).

### 3.4 miR‐20b participates in cardiac hypertrophy

**Figure 3:**
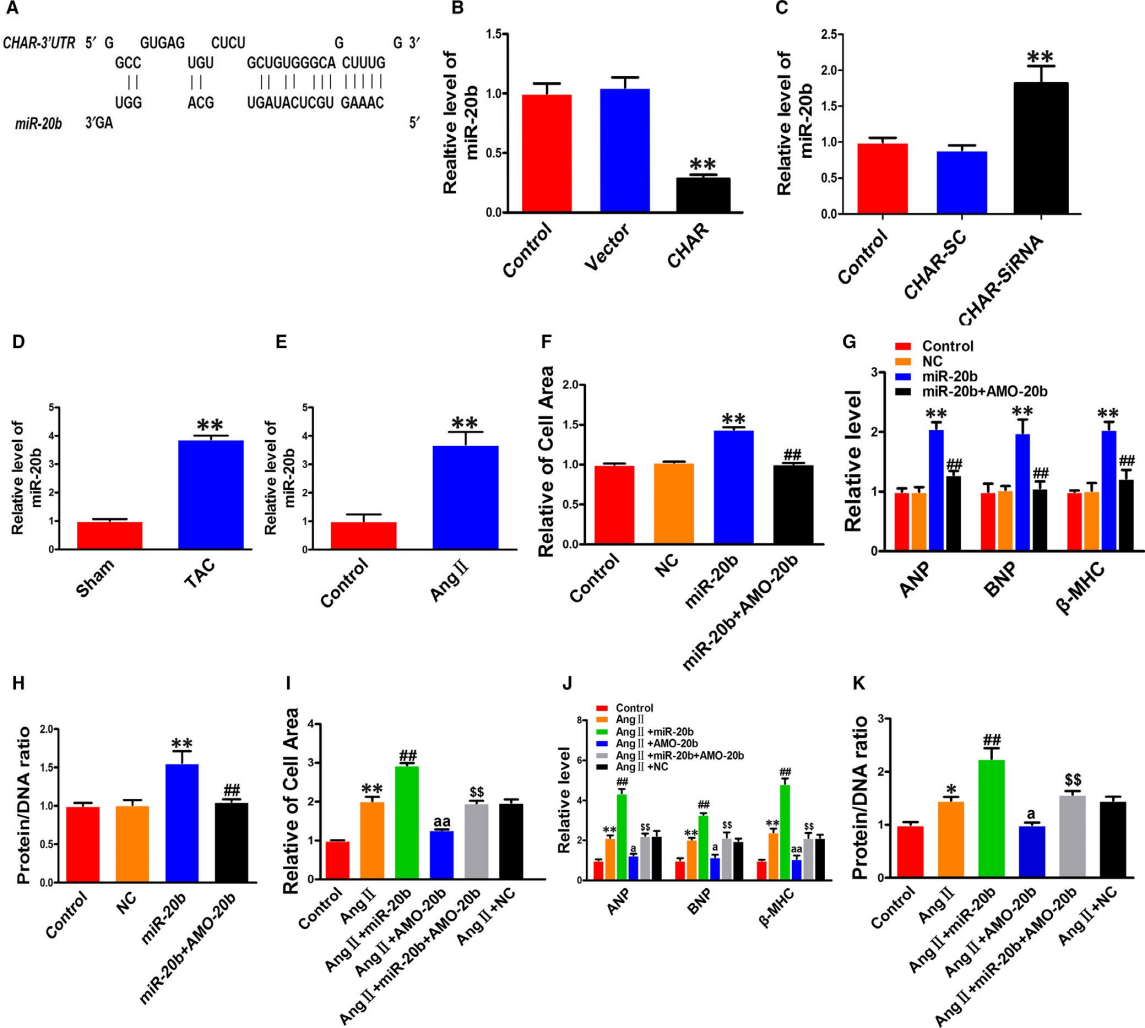
The pro‐hypertrophic effects of miR‐20b in NRVCs. A, Sequence alignment showing the complementarity between CHAR and miR‐20b. B, Overexpression of CHAR after transfection with CHAR‐plasmid decreased the level of endogenous miR‐20b, relative to the empty vector as a negative control in NRVCs. **P < 0.01vs Vector; n = 3. C, Knockdown of CHAR by siRNA increased miR‐20b level in NRVCs. CHAR‐SC: scramble RNA as a negative control. **P < .01 vs CHAR‐SC; n = 4. D, Up‐regulation of miR‐20b in TAC hearts relative to sham‐operated control mice. **P < .01 vs Sham; n = 4. E, Up‐regulation of miR‐20b in AngII‐treated NRVCs relative to non‐treated cells. **P < .01 vs Control; n = 4. F, Transfection of miR‐20b mimic increased cell size in NRVCs (n = 50). NC failed to affect the cell size, and co‐transfection of miR‐20b and AMO‐20b reversed the miR‐20b‐induced enlargement of cell size. G, Transfection of miR‐20b mimic increased the mRNA levels of ANP, BNP and β‐MHC in NRVCs (n = 4). NC failed to affect biomarkers, and co‐transfection with AMO‐20b reversed the miR‐20b‐induced increases. H, Transfection of miR‐20b mimic increased the protein/DNA ratio in NRVCs (n = 4). NC did not affect the ratio, and co‐transfection with AMO‐20b reversed the increase. **P < .01 vs Control (NC) and ^##^ *P *< .01 vs miR‐20b; I‐K, miR‐20b exacerbated, whereas AMO‐20b reversed the hypertrophic phenotypes in response to AngII stimulation in NRVCs, including the increased cell area (n = 50; I); mRNA levels of ANP, BNP and β‐MHC (n = 3; J); and protein/DNA ratio (n = 6; K). AMO‐20b abolished the effects of miR‐20b. *P < .05 & **P < .01 vs Control; ^##^ *P *< .01 vs AngII; ^aa^ *P *< 0.01 & ^a^ *p* < 0.05 vs AngII; and ^$$^ *P *< .01 vs miR‐20b + AngII. AngII, angiotensin II; ANP, atrial natriuretic peptide; BNP, brain natriuretic peptide; CHAR, cardiac hypertrophy‐associated regulator; NC, negative control; NRVC, neonatal rat ventricular cells; PTEN, Phosphatase and tensin homolog; β‐MHC, β‐myosin heavy chain

Long non‐coding RNAs can act as ceRNAs by absorbing miRNAs like a sponge through the sequence complementarity mechanism.^35^, ^36^, ^37^ To test whether CHAR modulated CH by the ceRNA mechanism, we performed the following analyses. First, we did computational analysis using the BiBiserv database to predict the miRNAs that have the potential to bind to CHAR. In this way, we identified miR‐20b as a candidate target for CHAR (Figure 3A). Second, overexpression of CHAR robustly reduced the level of miR‐20b, whereas silence of CHAR increased it (Figure 3B,C). We therefore focused our subsequent experimentation on CHAR and miR‐20b.

The level of miR‐20b was found markedly up‐regulated in both in vivo mouse model of CH induced by TAC and in vitro cellular model of cardiomyocyte hypertrophy induced by AngII in NRVCs (Figure 3D,E). The cells transfected with miR‐20b had a significantly larger cell size than those transfected with NC (Figure 3F). This hypertrophic response to miR‐20b was minimized after co‐transfection with AMO‐20b, the antisense inhibitor of miR‐20b (Figure 3F). Moreover, the hypertrophic marker genes ANP, BNP and β‐MHC, and the protein/DNA ratio were all increased in their expression by miR‐20b in NRVCs, and these effects were effectively reversed by AMO‐20b (Figure 3G,H). Furthermore, we transfected miR‐20b or NC into NRVCs treated with AngII. As depicted in Figure 3I‐K, miR‐20b exaggerated the hypertrophic response to AngII stimulation, as indicated by the enhanced hypertrophic phenotypes including enlarged cell surface area; up‐regulated expression of ANP, BNP and β‐MHC; and increased protein/DNA ratio. As anticipated, knockdown of miR‐20b by its inhibitor AMO‐20b abrogated all these hypertrophic phenotypes (Figure 3I‐K).

### 3.5 PTEN/AKT as a mediator of miR‐20b action

**Figure 4:**
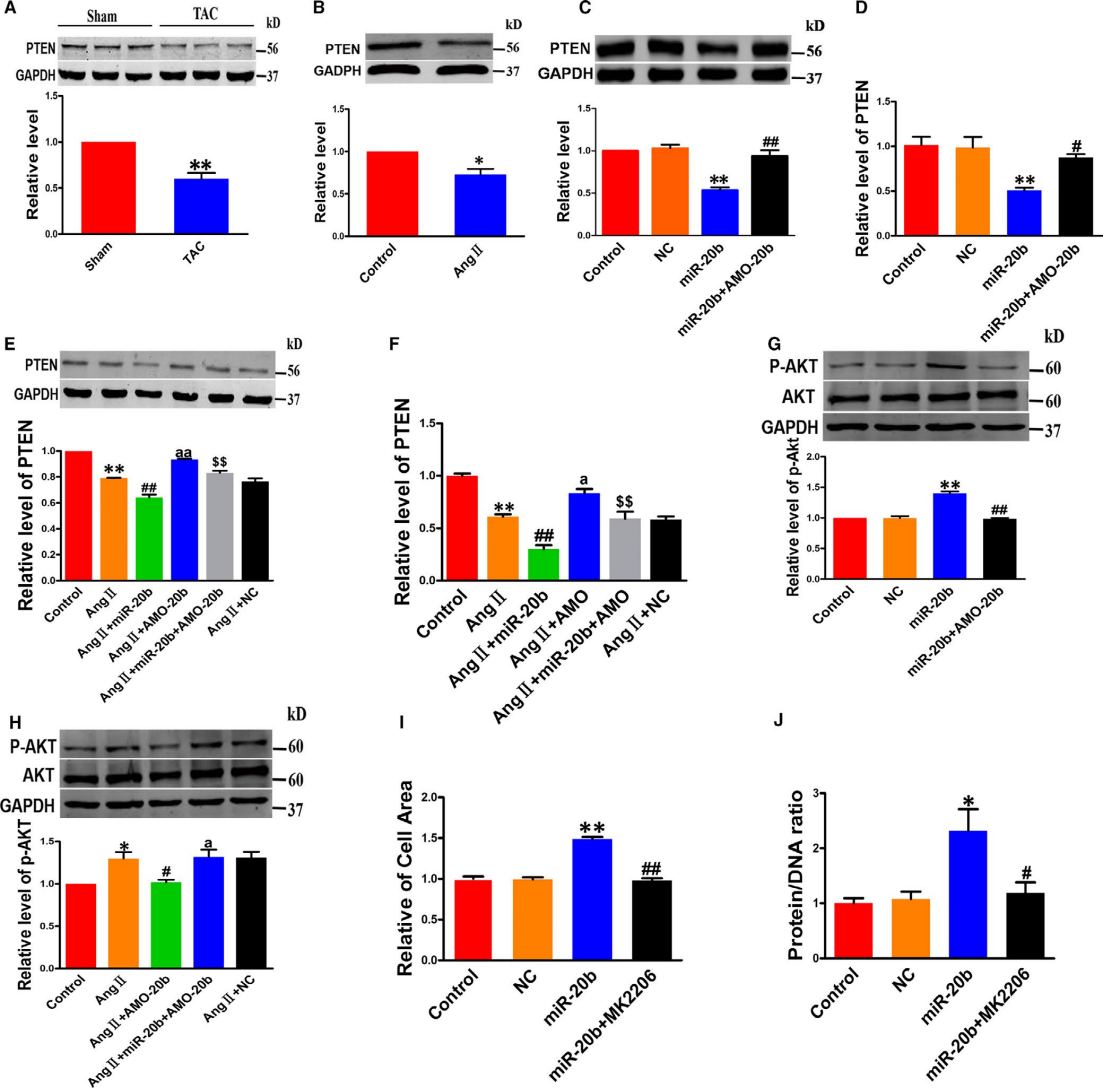
PTEN/AKT as downstream signalling molecules of miR‐20b action. A, Decrease in protein level of PTEN in TAC hearts compared to sham control hearts. **P < .01 vs Sham; n = 5. B, Decrease in protein level of PTEN in hypertrophic NRVCs induced by AngII compared to non‐treated cells. *P < .05 vs Control; n = 5. C, D, Repressive effects of miR‐20b on PTEN expression at both protein (n = 5) and mRNA (n = 4) levels. Co‐transfection with AMO‐20b reversed the effects of miR‐20b. **P < .01 vs Control (NC) and ^##^ *P *< .01 & ^#^ *P* < .05 vs miR‐20b. E, F, miR‐20b exacerbated, whereas AMO‐20b reversed the down‐regulation of PTEN at both protein (n = 4) and mRNA (n = 3) levels induced by AngII treatment. **P < .01 vs Control; ^##^ *P *< .01 vs AngII; ^aa^ *P *< 0.01 & ^a^ *p* < 0.05 vs AngII; and ^$$^ *P *< .01 vs miR‐20b + AngII. G, Effects of miR‐20b on the protein levels of total AKT (t‐AKT) and phosphorylated form of AKT (p‐AKT) in NRVCs. Note that miR‐20b up‐regulated p‐AKT without altering total AKT, whereas AMO‐20b reversed the effects. **P < .01 vs Control (NC) and ^##^ *P *< .01 vs miR‐20b; n = 7. H, The protein levels of total AKT and p‐AKT in NRVCs co‐transfected with miR‐20b mimic and AMO‐20b upon AngII treatment. *P < .05 vs Control; ^#^ *P < *.05 vs AngII; ^a^ *p < *0.05 vs AngII + AMO; n = 6. I & J, AKT inhibitor MK2206 reversed the increased cell area (n = 50) and protein/DNA ratio (n = 4) induced by miR‐20b. **P < .01 & *P < .05 vs Control (NC); and ^##^ *P *< .01 & ^#^ *P* < .05 vs miR‐20b. AngII, angiotensin II; NC, negative control; PTEN, Phosphatase and tensin homolog

Phosphatase and tensin homolog is a tumour‐repressive factor and has been reported to inhibit CH. Intriguingly, miR‐20b is known to regulate PTEN directly.^21^ In agreement with previous studies, our study revealed a significant decrease in PTEN protein level in TAC hearts and hypertrophic cardiomyocytes (Figure 4A,B). Similar to the hypertrophic stimulation, miR‐20b down‐regulated PTEN expression at both protein and mRNA levels in NRVCs without AngII treatment, and these effects were effectively reversed by AMO‐20b (Figure 4C,D). Consistently, AMO‐20b also mitigated the down‐regulation of PTEN induced by AngII (Figure 4E,F).

AKT is a downstream effector of PTEN, and it is involved in the regulation of CH. Our analysis showed that the total protein levels of AKT (t‐AKT) remained unaltered after miR‐20b transfection in NRVCs, whereas the active or phosphorylated form of AKT (p‐AKT) was significantly increased by miR‐20b (Figure 4G). When exposed to AngII, miR‐20b imposed further increase in p‐AKT without affecting t‐AKT (Figure 4H). AMO‐20b reversed the changes of p‐AKT caused by either miR‐20b transfection or AngII treatment (Figure 4G,H).

While the above results suggested that the PTEN‐AKT pathway is involved in the pro‐hypertrophic effect of miR‐20b, it remained uncertain whether AKT truly mediated the action. Our subsequent experiments using an AKT inhibitor MK2206 generated a piece of more conclusive evidence for the notion. As illustrated in Figure 4I,J, MK2206 diminished the hypertrophic responses induced by miR‐20b.

### 3.6 CHAR regulates cardiac hypertrophy via the miR‐20b/PTEN/AKT pathway

**Figure 5:**
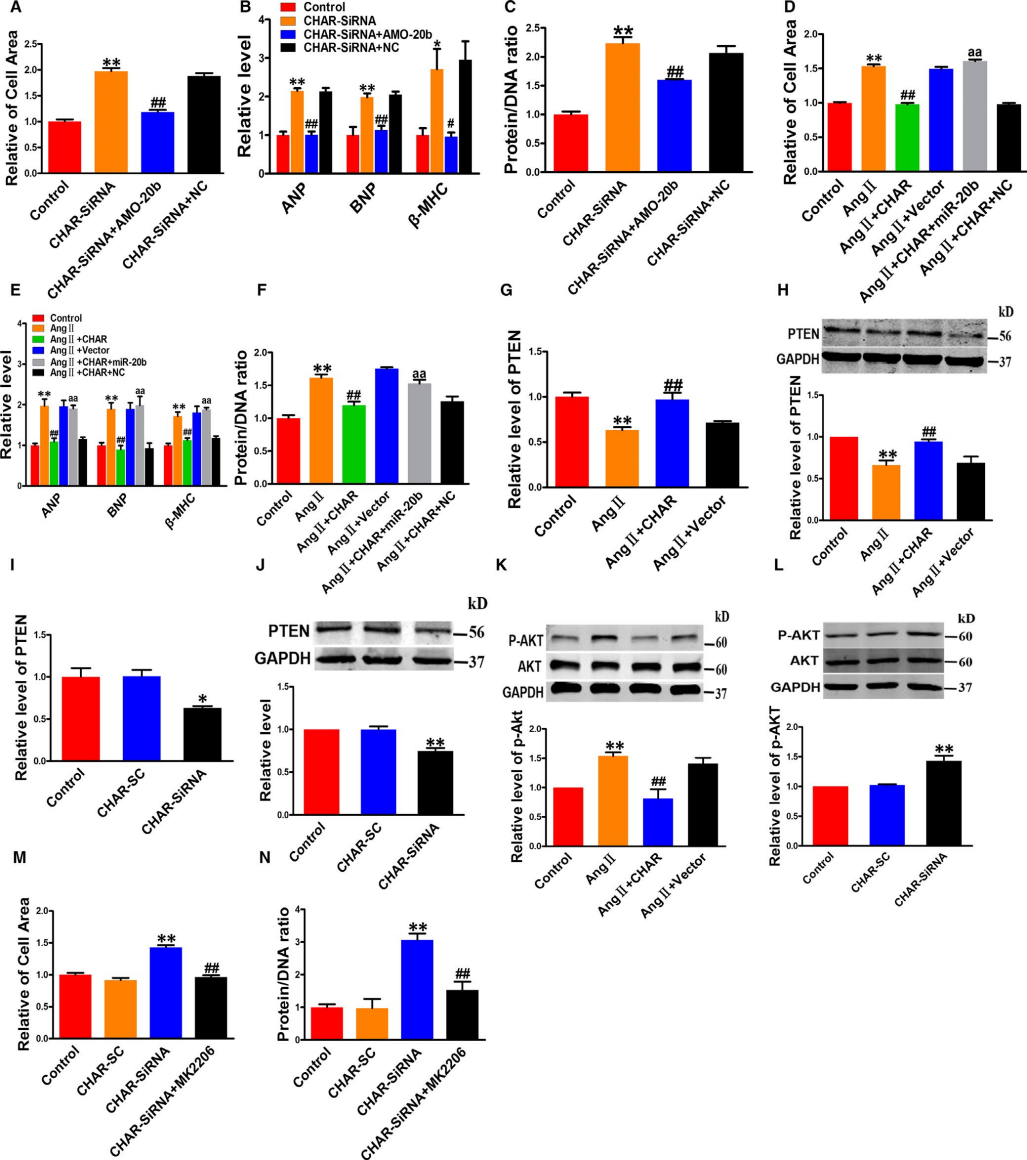
Roles of miR‐20b and PTEN/AKT in mediating the anti‐hypertrophic action of CHAR. A‐C, Knockdown of endogenous miR‐20b by AMO‐20b abrogated the pro‐hypertrophic effects of CHAR silencing by CHAR‐siRNA, as indicated by the changes of cell area (n = 50; A); ANP, BNP and β‐MHC mRNA levels (n = 4; B); and protein/DNA ratio (n = 4; C) in NRVCs. **P < .01 vs Control and ^##^ *P *< .01 & ^#^ *P* < .05 vs CHAR‐siRNA. D‐F, miR‐20b mitigated the anti‐hypertrophic effects of CHAR in NRVCs incubated with AngII, as indicated by the changes of cell surface area (n = 50; D); mRNA levels of ANP, BNP and β‐MHC (n = 4; E); and protein/DNA ratio (n = 4; F). **P < .01 vs Control; ^##^ *P *< .01 vs AngII; ^aa^ *P *< 0.01 vs AngII + lncRNA. G, H, CHAR countered the AngII‐induced expression down‐regulation of PTEN at mRNA (n = 3; G) and protein levels (n = 5; H) in NRVCs. **P < .01 vs Control; ^##^ *P *< .01 vs AngII. I, J, CHAR silencing down‐regulated PTEN expression at mRNA (n = 4; I) and protein levels (n = 6; J) in NRVCs, resembling the effects of AngII. **P < .01 & *P < .05 vs Control (SC). K, CHAR overexpression reversed the AngII‐induced up‐regulation of p‐AKT in NRVCs. **P < .01 vs Control; ^##^ *P *< .01 vs AngII, n = 6. L, CHAR silencing up‐regulated p‐AKT in NRVCs, resembling the effect of AngII. **P < .01 vs Control (SC); n = 6. M, N, AKT inhibitor MK2206 abolished the pro‐hypertrophic effects of CHAR silencing by siRNA as indicated by the change of cell area (n = 50; M) and protein/DNA ratio (n = 4; N). **P < .01 vs Control (SC), and ^##^ *P *< .01 vs CHAR‐siRNA. AngII, angiotensin II; ANP, atrial natriuretic peptide; BNP, brain natriuretic peptide; CHAR, cardiac hypertrophy‐associated regulator; lncRNA, Long non‐coding RNA; NRVC, neonatal rat ventricular cells; PTEN, Phosphatase and tensin homolog; β‐MHC, β‐myosin heavy chain

The results presented above provided evidence for the existence of a novel signalling pathway leading to hypertrophic phenotypes: CHAR↓ → miR‐20b↑ → PTEN↓ → p‐AKT↑ → hypertrophy. Yet, the link between CHAR and miR‐20b as well as the downstream components as a causal factor for hypertrophic development has not thus far been established. To this end, we conducted the following experiments. Co‐transfection of CHAR siRNA and AMO‐20b to prevent the increase in miR‐20b attenuated the hypertrophic responses induced by CHAR silence in NRVCs, as indicated by the reduced cell surface area; ANP, BNP and β‐MHC expression levels; and the protein/DNA ratio (Figure 5A‐C). On the other hand, overexpression of CHAR suppressed the AngII‐induced hypertrophic responses, while in the presence of miR‐20b, CHAR lost its ability to reverse the AngII‐induced up‐regulation of cell surface area; ANP, BNP and β‐MHC levels; and the protein/DNA ratio (Figure 5D‐F). We then showed that overexpression of CHAR resulted in up‐regulation of PTEN levels after AngII treatment (Figure 5G,H). In contrast, silence of CHAR attenuated PTEN expression at both mRNA and protein levels in vitro (Figure 5I,J; Figure S4A,B).

In agreement with the in vitro data, CHAR overexpression abrogated, whereas CHAR knockdown exacerbated the abnormal down‐regulation of PTEN protein level in TAC mice (Figure S5A,B). The negative control constructs failed to affect TAC‐induced down‐regulation of PTEN protein level.

We further observed that overexpression of CHAR decreased the level of p‐AKT, whereas knockdown of CHAR increased it (Figure 5K,L; Figure S4C). Consistently, the TAC mice with CHAR‐overexpression exhibited lower level of p‐AKT relative to vector‐treated counterparts, and CHAR silence increased the level of p‐AKT (Figure S5C,D). Furthermore, co‐transfection of CHAR‐siRNA and MK2206 significantly decreased the hypertrophic phenotypes in TAC mice (Figure 5M,N).

## 4 DISCUSSION

**Figure 6:**
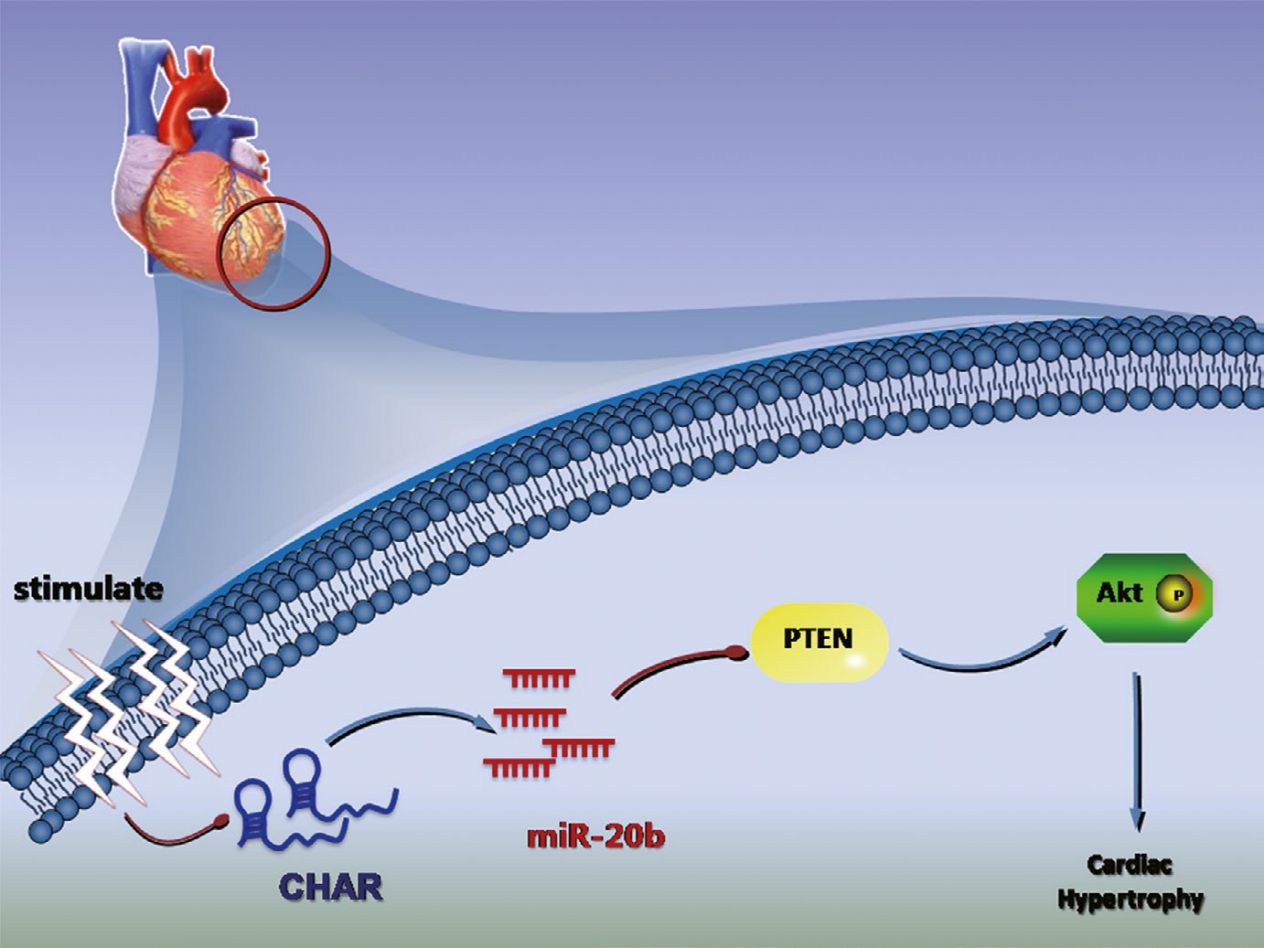
Schematic cartoon showing the proposed signalling pathway linking lncRNA CHAR to CH. lncRNA CHAR is down‐regulated in pathological CH; down‐regulation of CHAR weakens its ceRNA action on miR‐20b to increase the functional level of this miRNA; up‐regulation of miR‐20b represses its target gene PTEN leading to enhanced activation of AKT and consequent exaggeration of CH. This suggests a new signalling pathway: CHAR↓ → miR‐20b↑ → PTEN↓ → p‐AKT↑ → CH. CHAR, cardiac hypertrophy‐associated regulator; lncRNA, Long non‐coding RNA; PTEN, Phosphatase and tensin homolog

Pathological CH, characterized by diastolic and systolic disability and increased morbidity and mortality, is a major risk factor for HF, arrhythmia and sudden death. In the present study, we identified a lncRNA CHAR as a new regulator of CH. Specifically, we found that CHAR was an anti‐hypertrophic lncRNA and this lncRNA was markedly down‐regulated in CH induced by pressure overload created by transverse aortic constriction in vivo or by angiotensin II stimulation in vitro. We further elucidated the mechanism of action of CHAR as a ceRNA to sponge miR‐20b. Down‐regulation of CHAR resulted in an increase in the level of miR‐20b, enhancing direct repressive effect on its target gene PTEN, a known negative regulator of CH.^38^, ^39^ PTEN down‐regulation in turn promoted AKT activation to cause hypertrophic responses to pathological stimulation. Taken together, the present study identified a new lncRNA CHAR and unravelled a new pro‐hypertrophic signalling pathway mediated by CHAR: lncRNA‐CHAR/miR‐20b/PTEN/AKT (Figure 6). The findings should help advance our understanding of the cellular functionality and pathophysiological role of lncRNAs in the heart.

The concept of ceRNAs suggests that the RNA molecules that contain the binding sites (sequence complementarity) to a particular miRNA can compete one another by competitively binding to this individual miRNA to reduce its functional availability. lncRNAs can often act as ceRNAs, absorbing miRNAs through sponge‐like action. In such a way, lncRNAs indirectly regulate the target genes of miRNAs, or specifically, they release the target genes from repression of by the targeted miRNA.^40^ Indeed, a number of lncRNAs have been documented to regulate the development of cardiac disease through the ceRNA mechanism.^28^, ^29^, ^41^ The present study adds CHAR to the list of lncRNA‐ceRNAs. Our experiments showed that the level of miR‐20b was down‐regulated by CHAR overexpression but up‐regulated by CHAR silence. Such an inverse correlation between CHAR and miR‐20b expression suggests a targeting relationship. However, our luciferase assay showed that miR‐20b did not suppress the luciferase activity of CHAR (Figure S6). While the exact mechanism for this phenomenon is still unclear, it is possibly due to the incomplete complementarity between the seed site of miR‐20b and CHAR: there is an interruption of sequence complementarity within the seed region of miR‐20b. In addition, it is known that miRNAs act primarily by inhibiting protein translation with or without inducing RNA degradation. In our case, it is likely that miR‐20b does not cause lncRNA degradation.

MiR‐20b is one of the members of the miR‐17 family. Previous studies have shown that miR‐20b takes part in the regulation of cancers, including colon cancer, breast cancer, papillary thyroid carcinoma, glioblastoma phenotypes, gastric cancer and bladder cancer.^42^, ^43^, ^44^, ^45^, ^46^, ^47^ It has been reported that overexpression of miR‐20b increases apoptosis and promotes differentiation by activating the BMP signalling pathway in P19 cells. MiR‐20b impairs mitochondrial function by increasing the level of reactive oxygen species in P19 cells.^48^ The present study is the first to have identified the pro‐hypertrophic role of miR‐20b in the heart. MiR‐20b was up‐regulated in our hypertrophic animal and cellular models. Transfection of miR‐20b mimic into cardiomyocytes directly provoked the hypertrophic responses and promoted the hypertrophic phenotypes induced by TAC or AngII. Its inhibitor AMO‐20b effectively abrogated the pro‐hypertrophic action of miR‐20b.

Phosphatase and tensin homolog has been previously characterized as an anti‐hypertrophic signalling molecule.^38^, ^39^ Consistent with previous studies, significant decreases in PTEN at both protein and mRNA levels were confirmed in TAC hearts and hypertrophic cardiomyocytes. The PI3K/AKT pathway is the downstream components of PTEN and is one of the most important regulatory signalling pathways in CH and HF.^49^, ^50^ PTEN abolishes the AKT‐induced expression of GSK3β and p70S6K to inhibit CH.^51^ Our results demonstrated that changes of CHAR level were associated with the expression levels of PTEN and AKT. Overexpression of CHAR resulted in up‐regulation of PTEN levels and concomitant decrease in p‐AKT; vice versa, knockdown of CHAR attenuated PTEN expression with simultaneous increase in p‐AKT level.

Perhaps the most prominent finding of our study was the demonstration that CHAR overexpression could reverse the hypertrophic phenotypes in both in vivo animal model and in vitro cellular model. This result indicates that CHAR is an anti‐hypertrophic lncRNA and CHAR replacement might be an alternative strategy for the treatment of CH and the associated pathological alterations.

It must be noticed that in our model, expression down‐regulation of CHAR promoted hypertrophic responses of the heart (CHAR↓ → miR‐20b↑ → PTEN↓ → p‐AKT↑ → hypertrophy); yet the short‐term activation of AKT is protective to the heart via the inhibition of cell death, this would indicate a beneficial action of CHAR. Apparently, more rigorous investigations are needed to clarify this issue, and before that, this potential paradox is a limitation of the present study. Another limitation of our study is that we do not know the source of CHAR despite that our results indicate it is abundantly expressed in myocardium with Ct value of ~20. Rigorous future studies are needed to delineate the sources of this lncRNA. According to previous report, up‐regulation of miR‐19b inhibits apoptosis and activates the AKT pathway by down‐regulating PTEN.^52^ Here, we performed bioinformatics analysis using the RegRNA 2.0 database (http://regrna.mbc.nctu.edu.tw/html/prediction.html) to identify the miRNAs that contain potential binding sequences for CHAR. The database predicts no potential binding site or sequence complementarity between CHAR and miR‐19b. There is therefore unlikely existing any direct interaction between CHAR and miR‐19b.

## CONFLICT OF INTEREST

The authors declare no conflict of interest.

## AUTHOR CONTRIBUTIONS

Chaoqian Xu conceived the project, designed the experiments and edited the manuscript. Mingyu Zhang planned the experiments, integrated data and wrote the manuscript. Yuan Jiang, Xiaofei Guo and Bowen Zhang performed experiments, executed data analysis and wrote the manuscript. Jiangjiao Wu and Jiabin Sun performed animal studies and analysed the data. Haihai Liang, Hongli Shan, Yong Zhang, Jiaqi Liu_,_ Ying Wang, Lu Wang, Rong Zhang and Baofeng Yang provided vital reagents and technical support.
