# Supplementary material for: Long non‐coding RNA cardiac hypertrophy‐associated regulator governs cardiac hypertrophy via regulating miR‐20b and the downstream PTEN/AKT pathway
Source: J Cell Mol Med. 2019 Aug 29;23(11):7685–98. doi: 10.1111/jcmm.14641 (PMC6815784; doi:10.1111/jcmm.14641)
Supplement: Supplementary file 1 [file JCMM-23-7685-s001.docx]

**Supplemental Information**

**LncRNA CHAR Governs Cardiac Hypertrophy via Regulating miR-20b and the Downstream PTEN/AKT Pathway**

Running Title: Role of lncRNA CHAR in Cardiac Hypertrophy

Mingyu Zhang^2^*, Yuan Jiang^2^*, Xiaofei Guo^3^*, Bowen Zhang^2^, Jiangjiao Wu^2^, Jiabin Sun^2^_,_ Haihai Liang^2^, Hongli Shan^2^, Yong Zhang^2^, Jiaqi Liu^1^_,_ Ying Wang^1^, Lu Wang^4^, Rong Zhang^2^, Baofeng Yang^2^ and Chaoqian Xu^1,2^

^1^Center of Chronic Diseases and Drug Research of Mudanjiang Medical

University of Alliance of Sino-Russian Medical Universities, Mudanjiang

Medical University, Mudanjiang 157011, People’s Republic of China

^2^Department of Pharmacology (State-Province Key Laboratories of Biomedicine-Pharmaceutics of China, Key Laboratory of Cardiovascular Medicine Research, Ministry of Education), College of Pharmacy, Harbin Medical University, Xuefu Road 194, Harbin, Heilongjiang 150081, People’s Republic of China.

^3^Department of Pharmacy, the Second Affiliated Hospital of Harbin Medical University, No.246 Xuefu Road, Harbin, Heilongjiang Province, 150081, People's Republic of China.

^4^Department of Urology, the Fourth Hospital of Harbin Medical University

*These authors contributed equally to this work.

Corresponding author: Chaoqian Xu (chaoqx68@126.com)

**Supplementary Figures**

**Figure S1. Cardiac hypertrophy and cardiomyocyte hypertrophy models.**

**A.** Echocardiographic measurement of cardiac function indicating the successful establishment of CH. LVPWd: thickness of diastolic left ventricular posterior wall. **B** & **C.** Ejection fraction (EF) and fractional shortening (FS), respectively, showing the significant decreases of these two key functional parameters of the heart. **p*<0.05 & ***p*<0.01 *vs.* Sham; n=5. **D.** Increased ratio of heart/body weight (HW/BW) in the TAC mice relative to the sham group. ***p*<0.01 *vs*. Sham; n=5. **E.** Increased ratio of heart weight/tibia length (HW/TL) in the TAC mice relative to the sham group. **p*<0.05 *vs*. Sham; n=5. **F.** Hematoxylin and eosin staining (H&E; ×200 magnification) showing the enlarged cardiac muscles in TAC mice relative to sham counterparts. **G.** Upregulation of hypertrophic biomarker genes ANP, BNP, and β-MHC, assessed by quantitative real-time RT-PCR. **p*<0.05 & ***p*<0.01 *vs*. Sham; n=4 in each group. **H.** Immunofluorescence images (left panels; ×200 magnification) showing the enlarged cell size of neonatal rat ventricular cells (NRVCs) treated with angiotensin II (AngII) compared with non-treated cells. The right panel showing the changes of averaged data of cell surface area. Cardiomyocytes are identified by α-actinin antibody (red), and nuclei are stained with DAPI (blue). ***p*<0.01 *vs*. Control; n=50. **I.** Upregulation of mRNA levels of ANP, BNP, and β-MHC in NRVCs stimulated by AngII. **p*<0.05 *vs.* Control; n=4.

**Figure S2. Transfection of Test Constructs.**

**A.** Verification of CHAR overexpression in NRVCs transfected with the plasmid vector carrying the CHAR gene as compared to the empty vector as a control. ***p*<0.01 *vs*. Control (Vector); n=4. **B**. NRVCs were transfected with EGFP-labeled CHAR plasmid and the negative control for CHAR. Successful penetration of the labeled constructs into the cardiomyocytes indicated by the fluorescence signal in green ( cardiomyocytes were stained with α-actinin, nucleus were stained in blue with DAPI). **C.** Verification of the efficacy of CHAR-siRNA in knocking down endogenous CHAR in NRVCs relative to that of scramble negative control construct CHRF-SC. **p*<0.05 & ***p*<0.01 *vs*. Control ( SC); n=4. **D.** NRVCs were transfected with EGFP-labeled CHAR-SiRNA plasmid and the scramble control for CHAR. (cardiomyocytes were stained with α-actinin, nucleus were stained in blue with DAPI). **E.** The mice were injected through intra-cavity with lentivirus viral particles carrying CHAR or a vector for three weeks, and then were subjected to TAC operation for four weeks. The overexpression of CHAR in the mouse heart was verified. ***p*<0.01 *vs*. sham (Lenti-Vector); n=4. **F.** The lentivirus vectors carrying the short RNA for CHAR (sh-CHAR) or scrambled control RNA fragment (sh-Scr) were injected into mouse heart by intra-cavity for three weeks before TAC surgery. The specific knockdown of CHAR by sh-CHAR in the mouse heart was verified. ***p*<0.01 *vs*. Ctl (sh-Scr); n=4.

**Figure S3.** **Effects of CHAR on Cardiac contractile functionin hypertrophic heart.**

**A** & **B**. Increases in EF and FS by Lenti-CHAR for CHAR overexpression in TAC mice relative to sham-operated control mice. ***p*<0.01 *vs*. sham; ^##^*p*<0.01 *vs*. TAC + Lenti-Vector; n=6. **C** & **D**. Knockdown of CHAR by sh-CHAR decreases EF and FS induced by TAC; ***p*<0.01 & **p*<0.05 *vs*. sham; ^#^*p*<0.05 *vs*. TAC + Lenti-sh-Scr; n=5.

**Figure S4. Inhibition of CHAR induces hypertrophic responses in NRVCs in the presence of AngII.**

**A** & **B.** CHAR silence by siRNA exacerbates the AngII-induced downregulation of PTEN at mRNA (n=3; **A**) and protein levels (n=6; **B**). ***p*<0.01 *vs*. Control; ^##^*p*<0.01 *vs*. AngII; **C.** CHAR silence exacerbates the AngII-induced upregulation of p-AKT in NRVCs. ***p*<0.01 *vs.* Control; ^##^*p*<0.01 *vs*. AngII, n=7.

**Figure S5. Upregulation of protein levels of PTEN and p-Akt in response to hypertrophic stress and the regulation by CHAR.**

**A**. CHAR overexpression with Lenti-CHAR counters the TAC-induced expression downregulation of PTEN at the protein level. ***p*<0.01 *vs.* sham; ^#^ *p*<0.05 *vs*. TAC+Lenti-Vector, n=5. **B.** Knockdown of CHAR by sh-CHAR exacerbates the TAC-induced downregulation of PTEN at the protein level. ***p*<0.01 *vs.* sham; ^##^*p*<0.01 *vs.* TAC+Lenti-sh-Scr; n=5. **C.** CHAR overexpression with Lenti-CHAR reverses the TAC-induced upregulation of p-AKT in mice. **p*<0.05 *vs*. sham; ^#^*p*<0.05 *vs*. TAC+Lenti-Vector, n=5. **D.** Knockdown of CHAR by sh-CHAR exacerbates the TAC-induced upregulation of p-AKT in mice. **p*<0.05 *vs.* sham; ^#^*p*<0.05 *vs*. TAC+Lenti-sh-Scr, n=5.

**Figure S6.** **The luciferase activity.**

HEK293 cells were co-transfected with luciferase vector carrying wild-type CHAR sequence (CHAR-WT), miR-20b mimic or miR-NC. Dual luciferase activities were measured. n=3.
