# Supplementary figures and images for: Long non‐coding RNA cardiac hypertrophy‐associated regulator governs cardiac hypertrophy via regulating miR‐20b and the downstream PTEN/AKT pathway
Source: J Cell Mol Med. 2019 Aug 29;23(11):7685–98. doi: 10.1111/jcmm.14641 (PMC6815784; doi:10.1111/jcmm.14641)

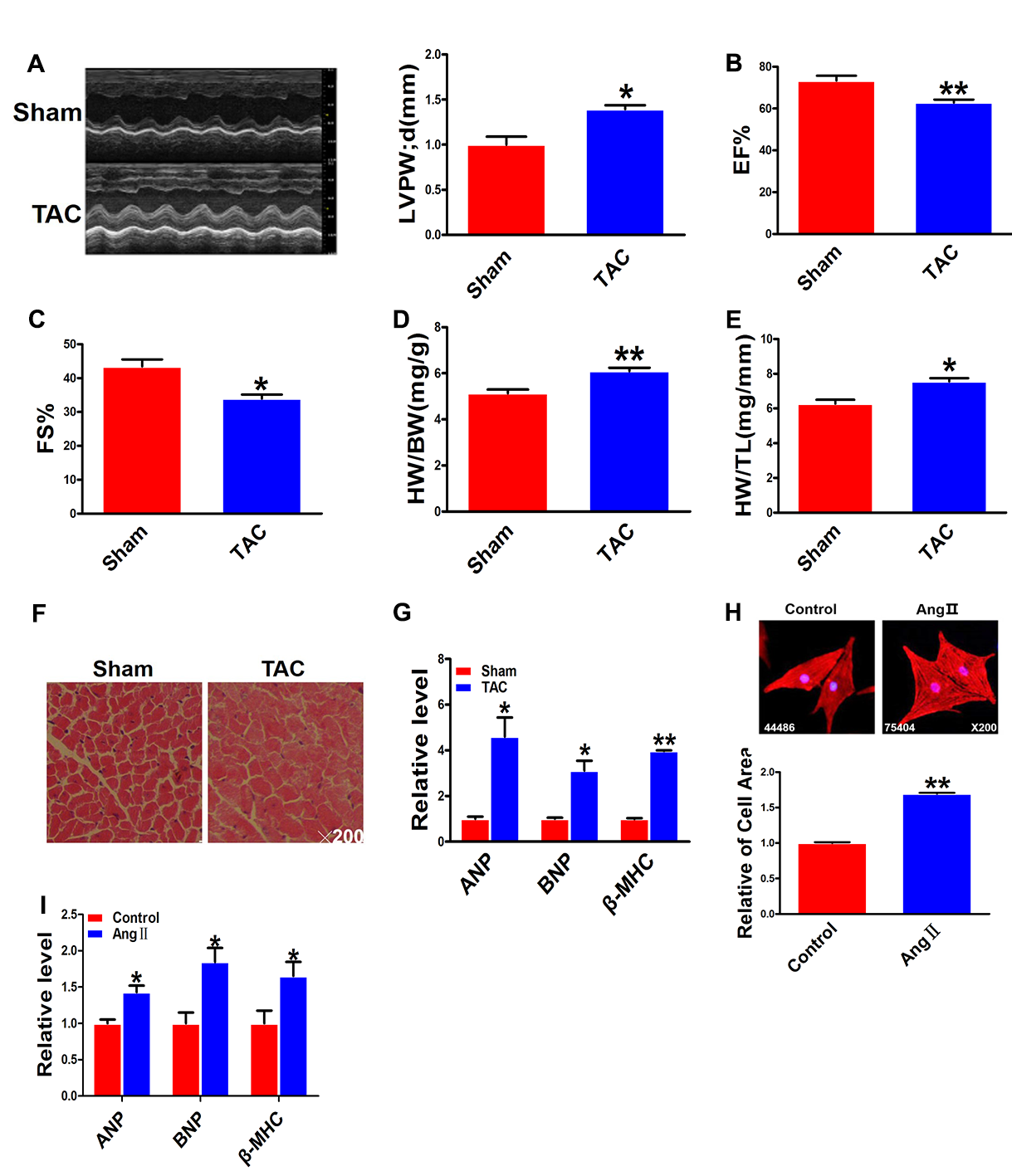

Supplement: Supplementary file 2 [file JCMM-23-7685-s002.tif]

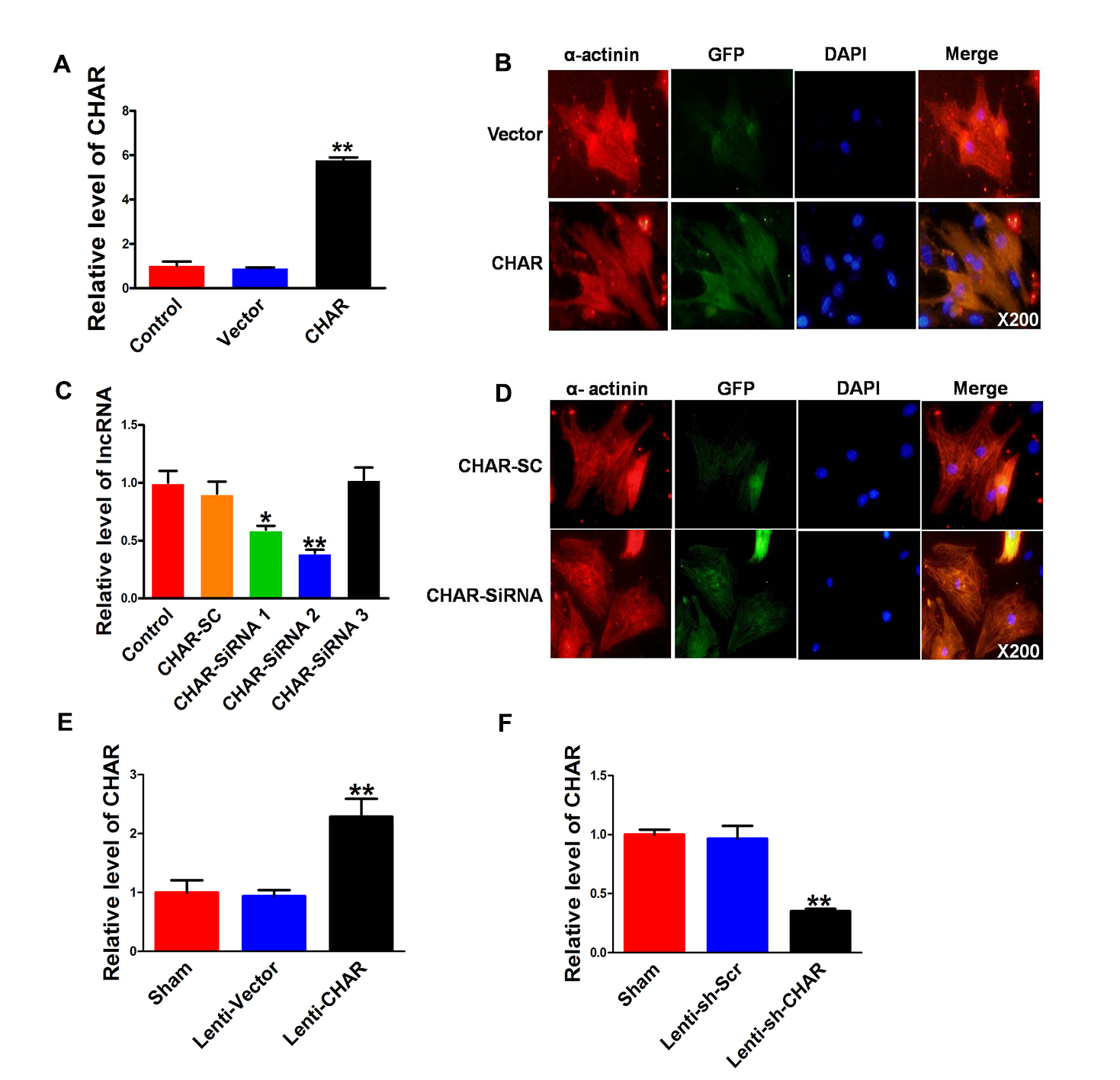

Supplement: Supplementary file 3 [file JCMM-23-7685-s003.tif]

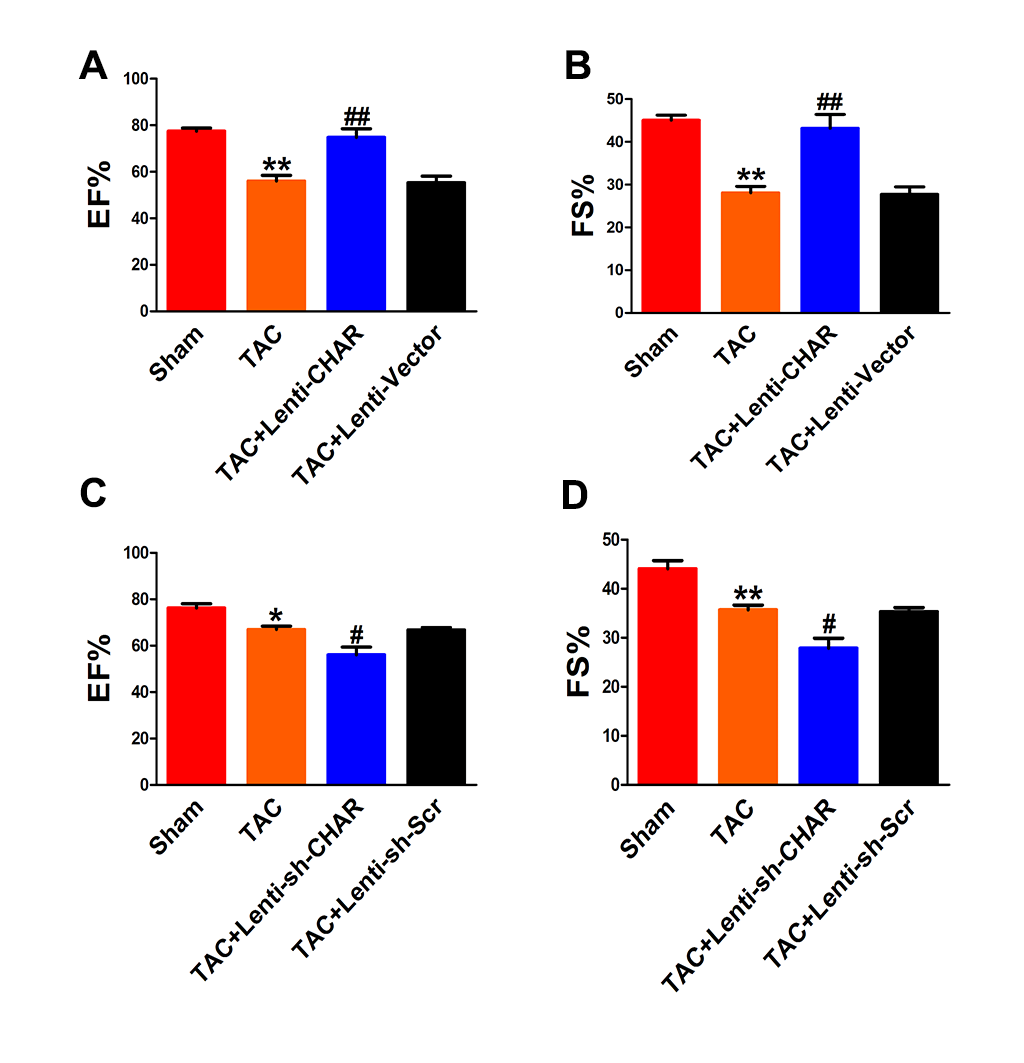

Supplement: Supplementary file 4 [file JCMM-23-7685-s004.tif]

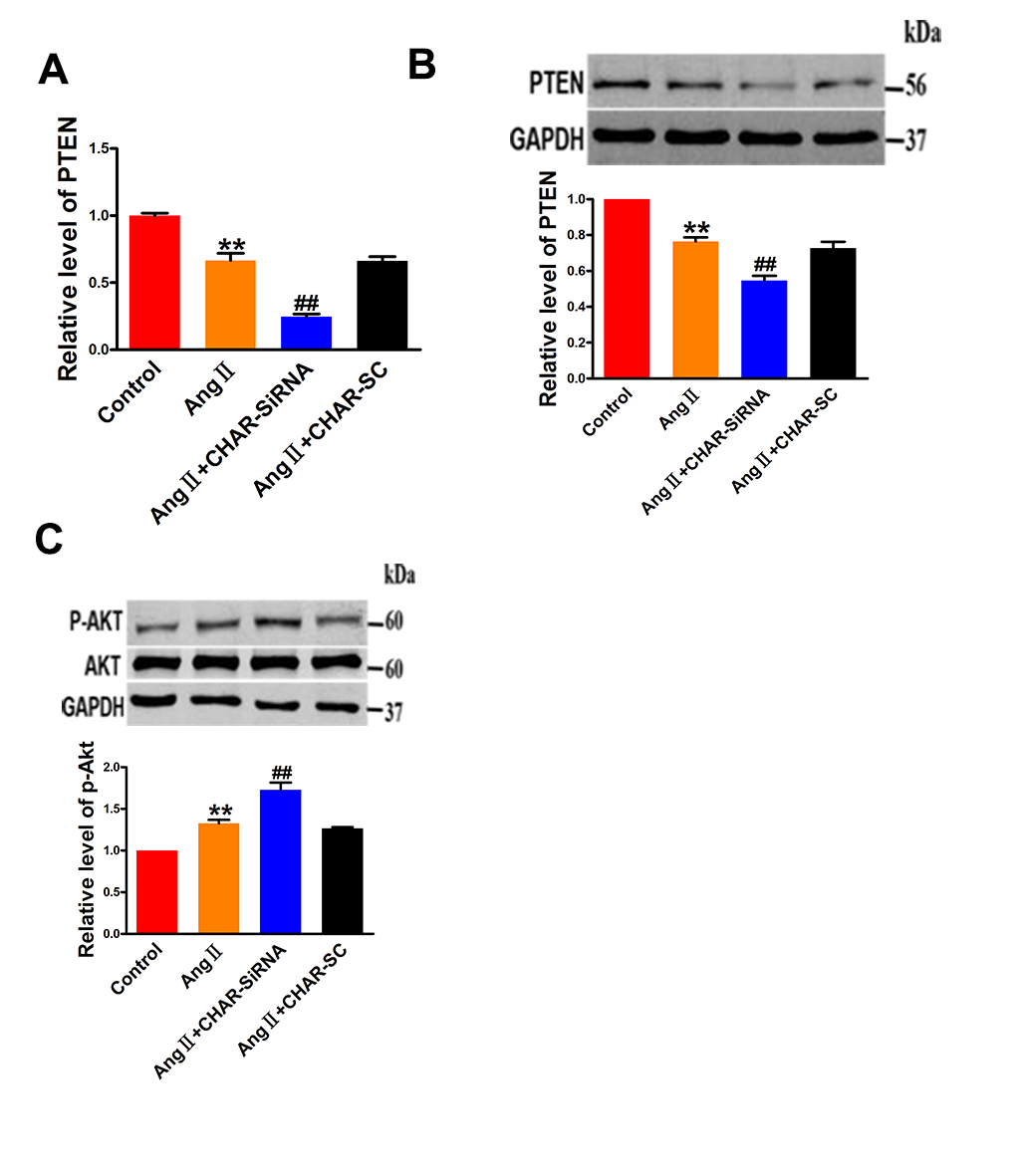

Supplement: Supplementary file 5 [file JCMM-23-7685-s005.tif]

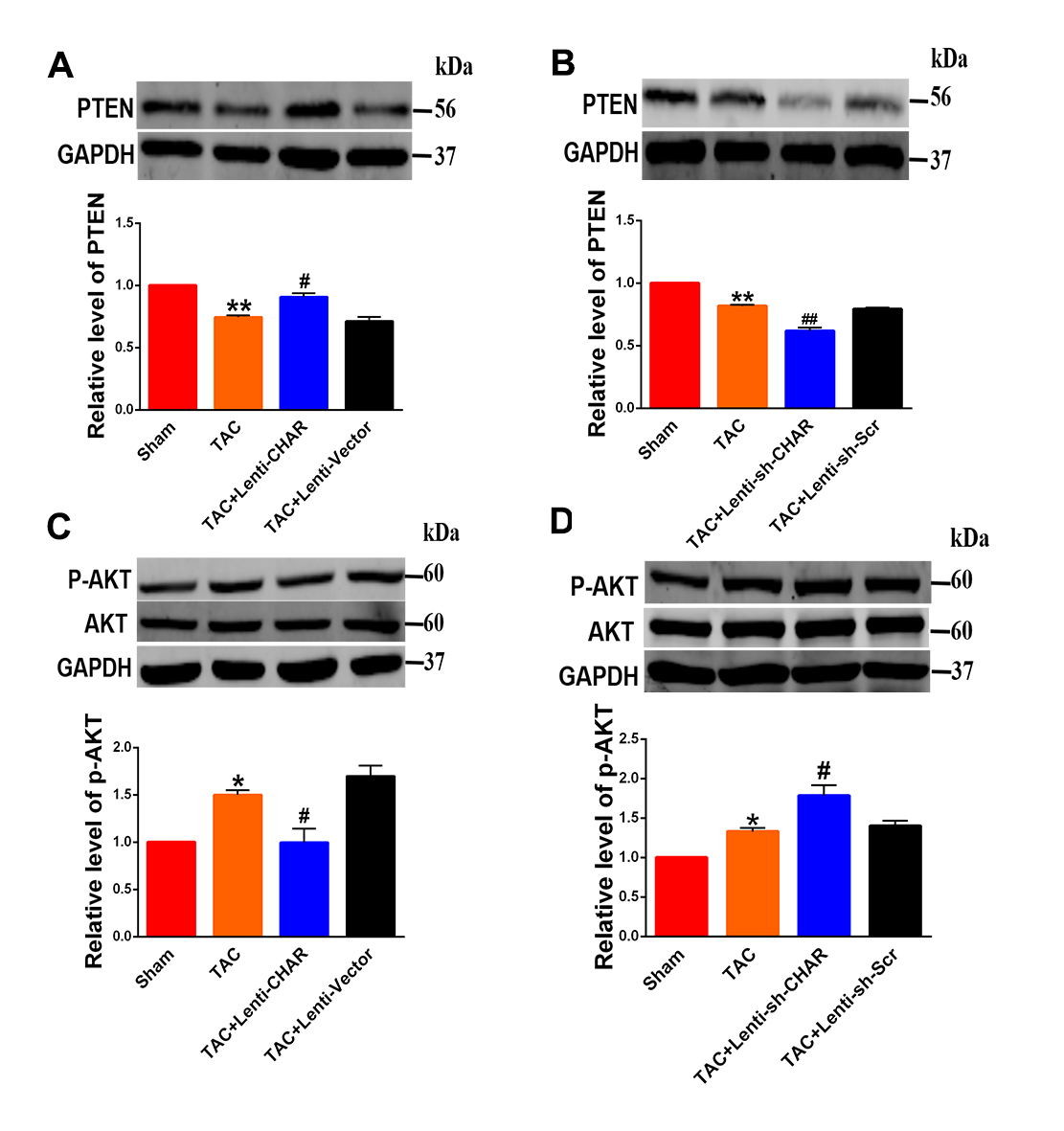

Supplement: Supplementary file 6 [file JCMM-23-7685-s006.tif]

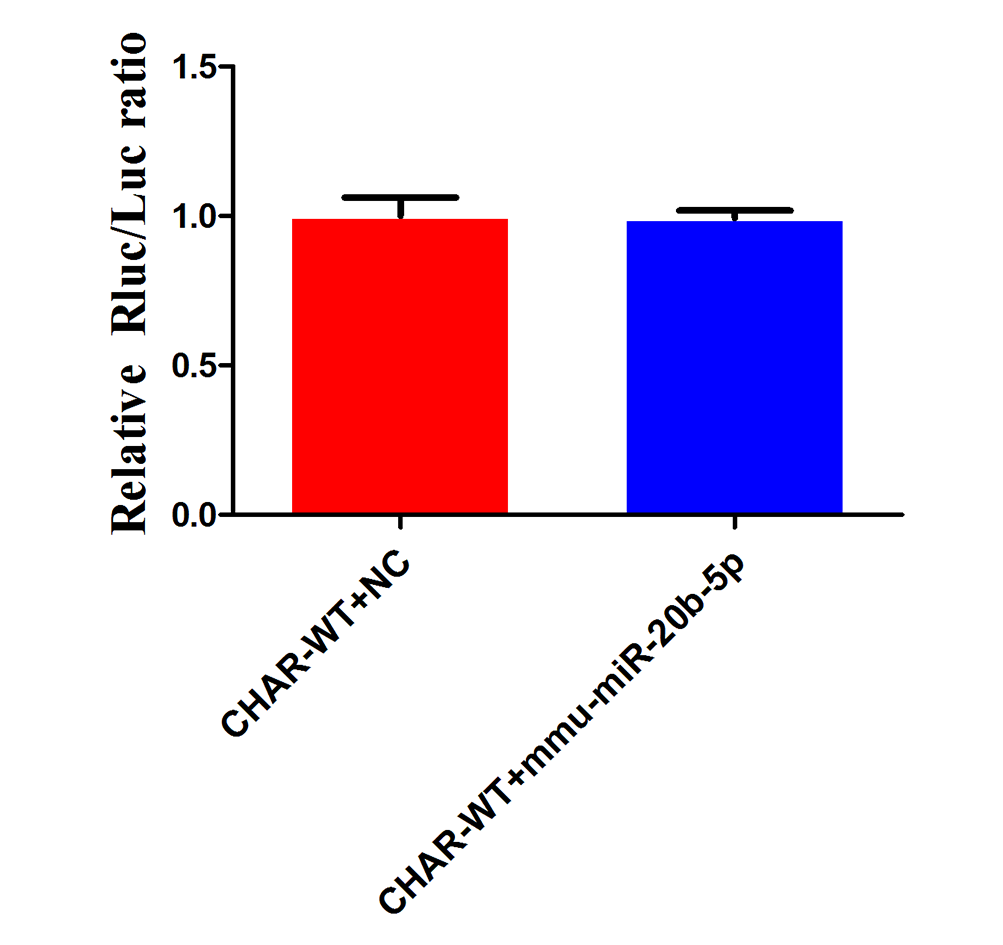

Supplement: Supplementary file 7 [file JCMM-23-7685-s007.tif]
