# Supplementary material for: Long non‐coding RNA cardiac hypertrophy‐associated regulator governs cardiac hypertrophy via regulating miR‐20b and the downstream PTEN/AKT pathway
Source: J Cell Mol Med. 2019 Aug 29;23(11):7685–98. doi: 10.1111/jcmm.14641 (PMC6815784; doi:10.1111/jcmm.14641)
Supplement: Supplementary file 8 [file JCMM-23-7685-s008.docx]

**



**

**Figure S7. Real-time RT-PCR analysis of miR-19b and CHAR. A**. Overexpression of CHAR by Lenti-CHAR does not affect the level of endogenous miR-19b, relative to the empty vector as a negative control. n=4. **B**. Knockdown of CHAR by shRNA does not influence miR-19b level. Scramble RNA was used as a negative control. n=4.


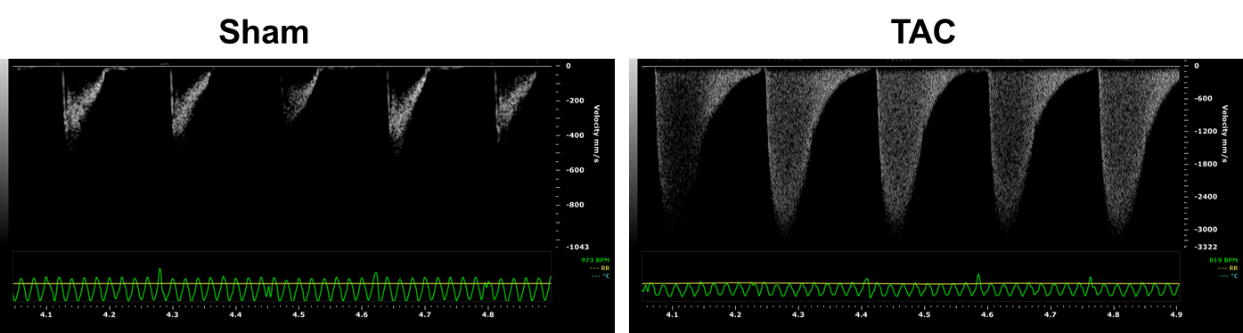





**Figure S8. The velocity of blood flow at the site of aortic constriction.** Comparison of blood velocity at the banding site of aorta between TAC mice and Sham control counteroarts. ***p*<0.01 *vs*. Sham; n=5.

**
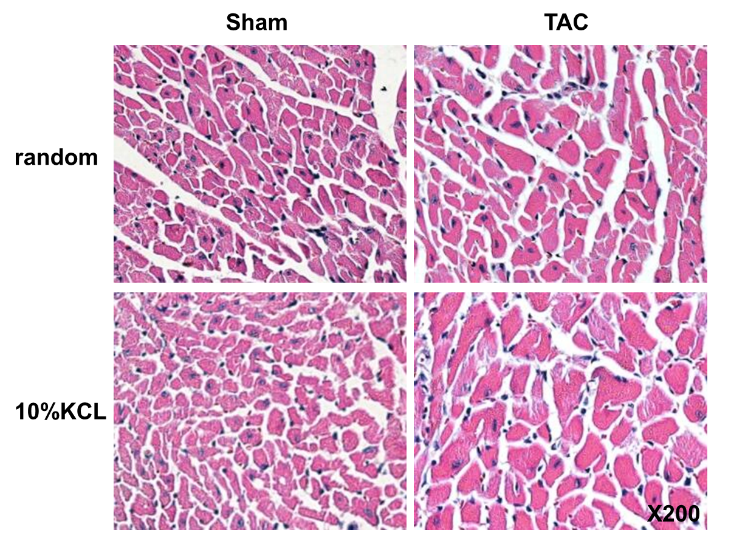
**

**Figure S9. HE staining of the cross-sectional area in 10% potassium chloride solution group and in the 4% paraformaldehyde group.**

HE staining showed that the cross-sectional area in 10% potassium chloride solution group was no significant difference compared with the 4% paraformaldehyde group both in sham mice and in TAC mice.
