# Supplementary material for: Long non‐coding RNA cardiac hypertrophy‐associated regulator governs cardiac hypertrophy via regulating miR‐20b and the downstream PTEN/AKT pathway
Source: J Cell Mol Med. 2019 Aug 29;23(11):7685–98. doi: 10.1111/jcmm.14641 (PMC6815784; doi:10.1111/jcmm.14641)
Supplement: Supplementary file 9 [file JCMM-23-7685-s009.docx]

**Supplementary Tables**

**Table S1. The velocity of blood flow at the site of aortic constriction.**

| Av Peak Vel (mm/s) | Sham | TAC |
| --- | --- | --- |
| 1 | -424.12 | -3340.78 |
| 2 | -467.89 | -3062.20 |
| 3 | -449.26 | -3075.30 |
| 4 | -549.81 | -3037.04 |
| 5 | -457.31 | -3215.81 |

**Table S2. Heart rate measurements with echocardiography (n=6).**

| **Parameter** | **Sham** | **TAC** | **TAC+Lenti-**  **CHAR** | **TAC+Lenti-**  **Vector** |
| --- | --- | --- | --- | --- |
| Heart rate（bpm） | 473.1±3.88 | 495.5±6.78 | 462.2±12.4 | 480.83±10.2 |
| **Parameter** | **Sham** | **TAC** | **TAC+Lenti-**  **sh-CHAR** | **TAC+Lenti-**  **sh-Scr** |
| Heart rate（bpm） | 472.3±15 | 491.7±4.9 | 483±7.8 | 471.7±14 |

**Values are mean ± SEM.**

**Table S3. The standard deviations of EF and FS.**

| **Standard deviations** | **Sham** | **TAC** | **TAC+Lenti-CHAR** | **TAC+Lenti-Vector** |
| --- | --- | --- | --- | --- |
| EF | 2.98 | 5.34 | 8.42 | 6.37 |
| FS | 2.72 | 3.35 | 7.25 | 3.87 |
| **Standard deviations** | **Sham** | **TAC** | **TAC+Lenti-sh-CHAR** | **TAC+Lenti-sh-Scr** |
| EF | 3.59 | 2.83 | 6.71 | 2.11 |
| FS | 3.32 | 1.87 | 4.07 | 1.68 |
